# Use of a Mobile App for the Process Evaluation of an Intervention in Health Care: Development and Usability Study

**DOI:** 10.2196/20739

**Published:** 2021-10-28

**Authors:** Winnie Szu Yun Chin, Alicia Kurowski, Rebecca Gore, Guanling Chen, Laura Punnett, SHIFT Research Team

**Affiliations:** 1 Center for the Promotion of Health in the New England Workplace (CPH-NEW) Lowell, MA United States; 2 Department of Public Health University of Massachusetts Lowell Lowell, MA United States; 3 Division of Population Sciences Dana-Farber Cancer Institute Boston, MA United States; 4 Department of Biomedical Engineering University of Massachusetts Lowell Lowell, MA United States; 5 Department of Computer Science University of Massachusetts Lowell Lowell, MA United States

**Keywords:** mobile apps, usability testing, user experience design, mobile phone, mhealth, iterative testing, participatory research, user demographics, worker participation

## Abstract

**Background:**

Process evaluation measures the context in which an outcome was or was not achieved through the ongoing monitoring of operations. Mobile apps are a potentially less burdensome tool for collecting these metrics in real time from participants. Research-driven apps are not always developed while paying attention to their usability for target users. Usability testing uncovers gaps in researchers’, developers’, and users’ mental models of what an efficient, effective, and satisfying product looks like and facilitates design improvement. Models may vary by user demographics.

**Objective:**

This study describes the development of a mobile app for collecting process evaluation metrics in an intervention study with health care workers that uses feedback at multiple stages to refine the app design, quantify usage based on workers' overall adoption of the app and the app's specific function, and compare the demographic and job characteristics of end users.

**Methods:**

An app was developed to evaluate the Center for Promotion of Health in the New England Workplace Healthy Workplace Participatory Program, which trains teams to develop solutions for workforce health obstacles. Labor-management health and safety committee members, program champions, and managers were invited to use the app. An accompanying website was available for team facilitators. The app’s 4 functions were meeting creation, postmeeting surveys, project time logs, and chat messages. Google Analytics recorded screen time. Two stages of pilot tests assessed functionality and usability across different device software, hardware, and platforms. In stage 1, student testers assessed the first functional prototype by performing task scenarios expected from end users. Feedback was used to fix issues and inform further development. In stage 2, the app was offered to all study participants; volunteers completed task scenarios and provided feedback at deployment. End user data for 18 months after deployment were summarized and compared by user characteristics.

**Results:**

In stage 1, functionality problems were documented and fixed. The System Usability Scale scores from 7 student testers corresponded to *good* usability (mobile app=72.9; website=72.5), whereas 15 end users rated usability as *ok* (mobile app=64.7; website=62.5). Predominant usability themes from student testers were *flexibility and efficiency* and *visibility of system status*; end users prioritized *flexibility and* *efficiency* and *recognition rather than recall*. Both student testers and end users suggested useful features that would have resulted in the large-scale restructuring of the back end; these were considered for their benefits versus cost. In stage 2, the median total use time over 18 months was 10.9 minutes (IQR 23.8) and 14.5 visits (IQR 12.5). There were no observable patterns in use by demographic characteristics.

**Conclusions:**

Occupational health researchers developing a mobile app should budget for early and iterative testing to find and fix problems or usability issues, which can increase eventual product use and prevent potential gaps in data.

## Introduction

### Background

In intervention research, process evaluation has become increasingly important to reliably assess the reasons for the effectiveness of an intervention or a lack thereof. Process evaluation is the ongoing monitoring of operations to measure the implementation process, and it provides a detailed context for subsequent outcomes evaluation [1]. When evaluated in occupational health interventions, this is often measured specifically as context, reach, dose delivered, dose received, fidelity, implementation, and recruitment [2]. Measuring these items requires data collection on the diversity of participants or organizations, recruitment or retention of members, their role in teams or activities, number and type of events attended, amount of time spent in and outside of the teams’ activities, benefits and challenges of participation, satisfaction with the work or process, and balance of power and leadership [1]. These research process activities can be hard to track during a participant’s workday or shift, and data collection might be more efficient if delivered through one medium.

The widespread use of smartphones has made mobile apps popular for mobile health (mHealth) studies, defined as “the use of mobile and wireless devices to improve health outcomes, health care services, and health research” [3]. However, there have been a limited number of tools developed to support and evaluate workplace improvement studies [4,5]. With the use of mobile apps, participant information can be collected in real time, increasing the convenience and, thus, ideally, participation level and data quality. Surveys can be created and sent at set intervals or during times where data collection is time sensitive and may be easier to incorporate into daily life [6]. Event logs can be entered at any time by participants to document where and when actions were taken by members [1]. With the flexibility of mobile apps, intervention activities and participant engagement and satisfaction can be tracked, and all of these are the primary components of process evaluation. Mobile apps provide a unique medium of data collection that might overcome the organizational and logistical barriers to data collection, which are common in occupational health studies.

Most mHealth apps are developed using a consumer-driven approach and are motivated by the participants’ perceived need to monitor their goals or manage their health condition. In contrast, apps created primarily for a research goal parallel a product-driven (or driving-markets) approach, which involves developing a unique product first and then influencing the structure or behavior of the existing market to gain a competitive edge [7]. These *research-driven* apps seek to fulfill a data collection need of the investigators [8] but may be at a disadvantage compared with consumer-driven apps, with regard to participants’ intrinsic motivation to use the app.

The usability of mobile apps strongly influences their actual use. User-centered design principles are recommended for mobile app development and include 4 principles: specify the context of use, specify app requirements, create design solutions in stages, and evaluate designs iteratively [9]. Ongoing evaluation through end user usability testing and quality assurance protocols is intended to enhance user satisfaction and uncover obstacles to effective and efficient product use. Unfortunately, the target users are infrequently involved in designing the features [10-12].

User-centered design is surprisingly difficult, and little empirical evidence has guided app development [4,12,13]. Empathy and appreciation for how users think and work are critical [13]. App development teams must not assume that users will approach the app in the same way that they would [13]. Participatory methods, such as card sorting, engage end users during the early development stages to design the information architecture to resemble users’ mental models [14,15]. This informs the development of prototypes that software developers often test on emulators. Real device testing is then needed to accommodate the various combinations of phone dimensions, screen resolutions, software versions, changing environmental contexts, and unreliable wireless networks that characterize mobile device interaction [16]. Testing with target users throughout the lifecycle of the app also helps with uncovering problems and discovering opportunities to improve the product while ensuring that the design is still flexible [17]. However, in the work context, end users may not have adequate time to devote to iterative and participatory design [15,18,19]; therefore, testing basic functionality in a nonrepresentative sample may be necessary to identify bugs before introducing the app to the user population. When usability testing is implemented only in later stages, fixes or feature requests are likely to be more costly and time consuming, as much of the structure has already been set [20]. Furthermore, research participants encountering early difficulties may become permanently discouraged from using the app throughout the study.

Some guidelines have been created for the iterative usability testing of mobile apps, but the form and extent of testing vary among studies, and validated instruments are not always used [21]. Laboratory-based testing is often tedious and expensive and has been criticized for not reflecting real use cases [22]. Others have proposed toolkits that can be embedded into the code of the mobile app to track user interface events from users [22]. A recent review suggested that combination approaches would be most useful [21]. Regardless of the method, testing with just 5 users helps identify 80% of the usability problems [23]. Information on usability testing should be documented as it has an impact on the adoption and use of the app [24].

Understanding relevant user demographics is important to assist in designing for a wide variety of target users [11]. The influence of user demographics on app use is not clear, particularly in mHealth studies. In one study focusing on a diabetes mHealth system, younger users performed app tasks faster and had fewer errors [11]. Another study on a cardiovascular disease risk management app found that younger populations downloaded the app more often, but older populations demonstrated greater sustained engagement [25]. Younger age groups may find mobile technologies commonplace and readily acceptable, but older adults are also interested in technology and are capable of acquiring complex computer skills [26]. Relationships between age and use of computer software have been examined in the literature, with some authors finding inverse relationships, whereas other authors did not find inverse relationships [27].

A US survey revealed that those who were younger, had more education, reported excellent health, and had higher income were the main users of health apps [28]. Another study reported that the odds of users downloading health apps were higher in college or graduate school than in high school and decreased with increasing age [29]. The existing literature provides some information regarding the type of users that use mHealth apps to improve their own health, but these differences may or may not apply to research-driven applications, including program evaluation.

### Objectives

The aforementioned gaps in the literature suggest that our mobile app is one of the very few apps developed to collect and conduct process evaluation in a participatory workplace change study. The aim of this study is to describe a user-centered development approach for a mobile app that tracks the process of a participatory intervention. In particular, this study seeks to (1) describe the iterative development of a mobile app to track a workplace change process, (2) identify functions most often used within the app by target users, and (3) examine demographic and job-related characteristics of app users.

## Methods

### Study Design

This descriptive study involved 5 health care facilities in the northeast United States participating in the Safety and Health through Integrated, Facilitated Teams (SHIFT) intervention study **(**Clinicaltrials.gov NCT04251429) [30]. SHIFT uses the Center for Promotion of Health in the New England Workplace Healthy Workplace Participatory Program (HWPP), a process for increasing the effectiveness of occupational health and safety committees through root cause analysis, identifying health and safety needs, and proposing solutions to leadership for implementation [31]. The joint labor-management Design Teams (DTs) are groups of 8 to 12 frontline workers from various departments, with 2 cofacilitators who chair the meetings and facilitate the HWPP process. The Steering Committee (SC) included upper-level managers responsible for budget and resource allocation. This usability study was conducted to guide the development of a data collection tool used in the trial; the number of subjects did not correspond to the anticipated enrollment for the trial itself.

### System Development

#### Mobile App

**Figure 1:**
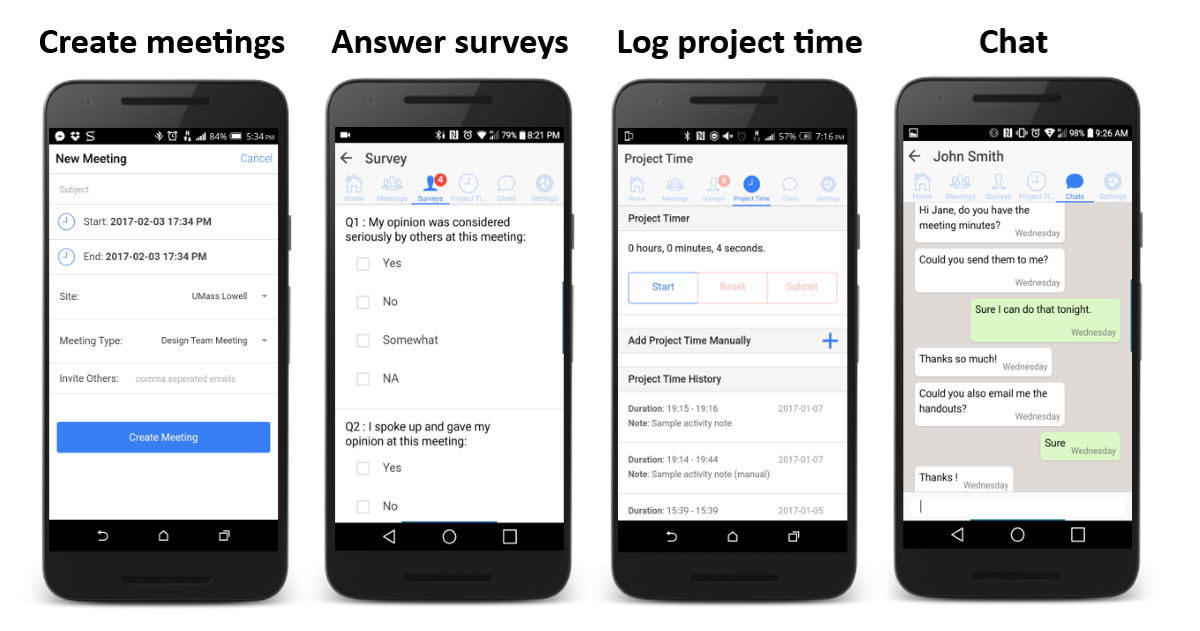
Safety and Health through Integrated, Facilitated Teams project mobile app: HWPP Assistant functions. HWPP: Healthy Workplace Participatory Program.

The HWPP Assistant app was developed for iOS (version 8.0 or higher) and Android (version 4.1 or higher) platforms using an agile approach. Detailed specifications were developed by the researchers in consultation with a computer scientist. The app had 4 main functions that allowed users to (1) create meetings, (2) answer surveys, (3) log time spent on project-related tasks, and (4) converse privately or with the entire group regarding any questions or concerns (Figure 1).

The meeting function provided the ability to create and view upcoming meetings. The 3 meeting types were DT, SC, and small group meetings. Cofacilitators could set up DT meetings, which automatically invited all DT members and cofacilitators at the specified site. SC members could create SC meetings. Anyone could call small group meetings by entering the email addresses of the desired meeting attendees. Once meetings were created, the invited users were able to see a list of upcoming meetings and download agendas attached to them. Users were reminded of meetings 24 hours and 2 hours before the meeting date or time via push notifications.

Surveys were sent at the end of meetings to all members, with specific questions based on their role in the study, intervention phase, and intervention status (control or intervention). Survey question templates were created by the research team and uploaded onto the website, where the majority of the administrative tasks were performed.

Time spent on project-related tasks was reported in either of the 2 ways: by using a start or stop timer, before or after executing the task, or by selecting a predetermined time interval of 30, 60, 90, or 120 minutes. The second method was designed as a backup in case the user forgot to use the timer or was not able to use their phone during the task. Both methods require an activity note to be submitted, describing the task executed.

Finally, the chat function allowed the users to chat privately or broadcast a message to users within their team. Private and broadcast chats included a timestamp, and push notifications were sent when a message was received.

An integrated page timer in the background recorded the time that users spent on each page function when the app was open and reported the information to Google Analytics. The app was included in the study protocol approved by the University of Massachusetts Institutional Review Board (approval number: #16-131-PUN-XPD).

#### Website Application

**Figure 2:**
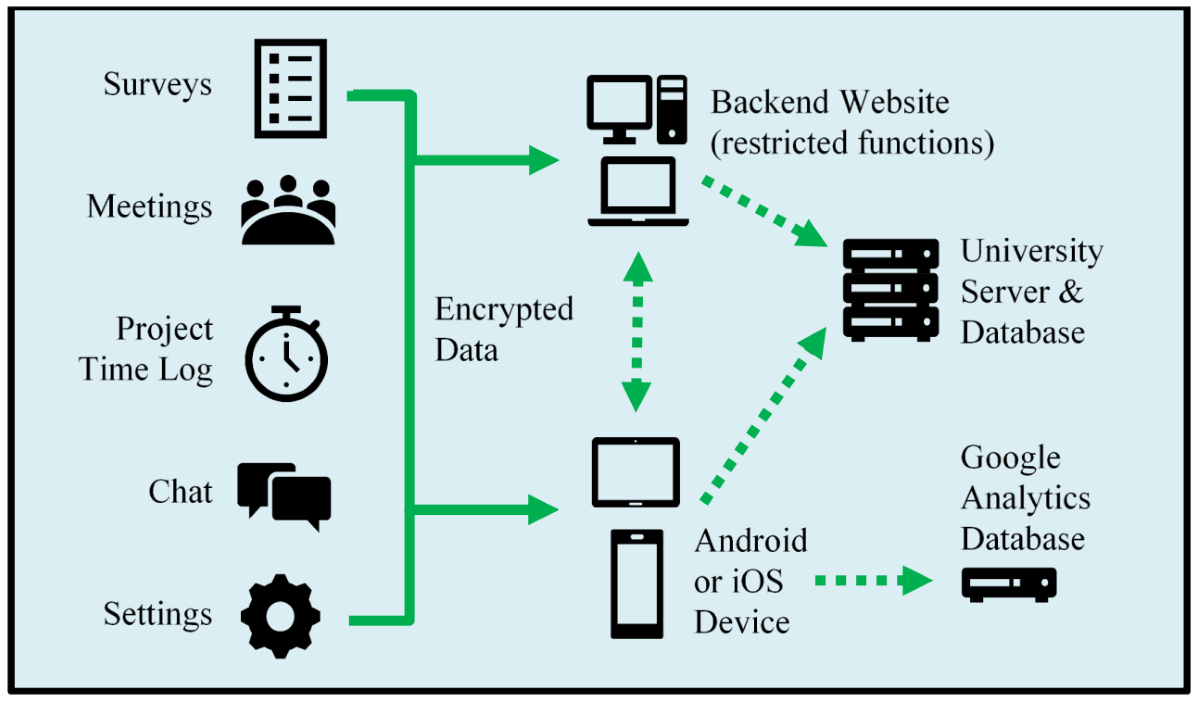
System architecture of the Safety and Health through Integrated, Facilitated Teams project mobile app HWPP Assistant. HWPP: Healthy Workplace Participatory Program.

Researchers entered users into the system through the accompanying website and monitored the incoming data in real time. Cofacilitators could set meeting times and upload meeting minutes and agendas using the website as well. All data were encrypted and sent to the back-end password-protected server hosted on the University of Massachusetts Lowell Department of Computer Science server (Figure 2) [32].

### Usability Testing

#### Stage 1: Student Testing

Initial testing of the app and accompanying website was done by 7 students because our formative research indicated that our target participants were overburdened with work demands. These undergraduate and graduate students were employed in the SHIFT project and were compensated for their time. Each student was assigned 1 to 6 roles to test. These roles were DT member, facilitator, SC member, champion, researcher, and administrator. Students were asked to provide informed consent and were instructed to think aloud while completing tasks, while the researcher observed the tester and took notes. A user guide was made available during the test. Once the student completed all the app tasks for one role, the researcher changed their role. This process was repeated for each student until all tasks were performed for the assigned roles. Depending on the students’ availability, some students had the option of testing when they were not observed, but they were asked to provide detailed descriptions and schedule a follow-up meeting if their feedback needed clarification.

The task scenarios were created based on the guidelines by Dumas and Redish [33] to mimic the functionality expected from the app across roles. Task scenarios were selected and developed based on (1) tasks that users would do with the product, (2) tasks that probed potential usability problems, and (3) tasks suggested from concerns and experience from initial testing by research team members [33]. Paper task scenarios were provided at the app pilot, where users were asked to perform the essential functions within the app and provide qualitative feedback. Further questions for each task included whether users encountered errors, whether they were able to complete the task, and whether they could see a more effective method to complete the task. Some roles had website tasks associated with them; this paper focuses on the app tasks.

At the end of the entire testing period, students answered the System Usability Scale (SUS) separately for the mobile app and the website [34]. The themes and issues were identified and reported back to the developer for the next iteration of the app.

#### Stage 2: End User Testing

The mobile app was deployed at all 5 sites among 94 participants who were engaged in the DTs and SCs at their respective sites. A walk-through tutorial was presented in person to all participants, along with hard-copy task scenarios and SUS forms for real-time evaluation. If necessary, users were allowed to complete task scenarios and SUS at home and mail them or return their evaluations at the next meeting.

### Demographics Survey and Team Roster

As part of the larger SHIFT project, surveys were distributed to all employees at the 5 sites. Demographic information was collected by these surveys and added to a team roster with other observationally collected information from these meetings. Information from these sources provided demographic and occupational information on the subgroups of participants in this study. This information was combined with end user usability responses using individuals’ randomized ID.

### Data Management and Statistical Analysis

All task scenarios and SUS surveys were entered into the project database. In stage 2, the app data were exported via the website and Google Analytics. Surveys distributed, surveys answered, meeting dates or time, chat sessions, project time logs, and app screen time were compiled for each end user for 18 months after deployment to SHIFT study end users.

Data were stored on an encrypted, password-protected drive in the Computer Science department at the University of Massachusetts Lowell. Backups from the back-end server and Google Analytics were run in parallel on the SHIFT project’s shared drive at 1-month intervals, which was the frequency expected for meetings and their associated surveys.

Usability scores were computed using the SUS scoring system [34]. SUS responses were scored from 0 to 100 and compared with a threshold of 68 and an adjective scale [35,36]. The NVivo 12 program (QSR International) was used to analyze the themes of qualitative feedback on the types of errors reported and fixed. One research assistant analyzed the feedback content by sorting through responses by the app’s functions and interpreting whether the feedback provided was focused on usability or functionality. Unique usability feedback was categorized using the usability heuristics developed by Nielsen [37]. In the cases where suggestions from testers and users could not be implemented, suggestions were documented and (where possible) alternative solutions were proposed.

SAS version 9.4 (SAS Institute) was used to analyze use and demographic information. The Kruskal-Wallis test and Wilcoxon rank sum tests were used to compare median screen time between groups based on demographic and job characteristics.

## Results

### Overview

The first prototype was created in November 2016. Pilot testing was carried out on versions 0.1.0 to 0.3.2 from December 2017 to February 2018. Three student testers used an iPhone with iOS operating system of 8.1.3-11.1.2, whereas the other 4 used Samsung smartphones with Android operating systems 4.4.2-7.0. Screen sizes and resolutions ranged from 4 to 5.7 inches and 540×960 to 2560×1440 pixels, respectively. Testing time ranged from 15 minutes to 5 hours per person, depending on the number of roles that each student tested and whether the test was moderated or unmoderated.

The app was deployed in June 2018. Approximately one-fourth (23/95, 24%) of the invited end users downloaded the app for use during the SHIFT project. Most of the use was by participants while they were in the intervention period. Most of the users were female (15/23, 65%); were White (20/23, 87%); were not Latino or Hispanic (19/22, 86%); reported their health as “very good” (8/14, 57%); were members of a union (16/22, 73%); worked the day shift (21/23, 91%); reported an income of at least US $75,000 (9/14, 64%); and had at least a college or professional degree (10/14, 71%; Table 1). The median total use time over 18 months was 10.9 minutes (IQR 23.8). The median total number of page visits was 14.5 visits (IQR 12.5). There were no significant differences in the median total use time and page visits between the demographic groups. When compared with those who did not use the app, app users were more likely to have college or professional education and to earn US $75,000 or more.

The tested app versions ranged from 1.0.1 to 1.0.5. Most (18/23, 78%) of the end users had Apple devices with iOS versions from 9.3.2 to 12.1.2, whereas 22% (5/23) were Android users with operating systems ranging from 7.0 to 9.0. Screen sizes and resolutions ranged from 5 to 6.4 inches and 1280×720 to 2880×1440 pixels, respectively.

**Table 1:** Demographic and job characteristics of Safety and Health through Integrated, Facilitated Teams participants who downloaded the HWPP^a^ Assistant app (n=23).

Characteristics		Participant^b^, n (%)
**Age group (years)**		
	25-39	10 (43)
	40-54	8 (35)
	≥55	5 (22)
**Sex**		
	Male	8 (35)
	Female	15 (65)
**Race**		
	White	20 (87)
	Unknown	3 (13)
**Ethnicity**		
	Latino or Hispanic	3 (14)
	Not Latino or Hispanic	19 (86)
**BMI**		
	Normal	2 (15)
	Overweight	6 (46)
	Obese	5 (38)
**Self-reported health**		
	Excellent	1 (7)
	Very good	8 (57)
	Good	4 (29)
	Fair	1 (7)
**Union status**		
	Member	16 (73)
	Nonmember	6 (27)
**Shift**		
	Day	21 (91)
	Evening	2 (9)
**Job title**		
	Administration	9 (39)
	Clinical	9 (39)
	Other	5 (22)
**Income (US $)**		
	25,000-49,999	2 (14)
	50,000-74,999	3 (21)
	≥75,000	9 (64)
**Education**		
	College or professional	10 (71)
	Postgraduate	4 (29)

^a^HWPP: Healthy Workplace Participatory Program.

^b^Missing information is excluded.

### Stage 1: Student Testing

#### Overview

The average SUS scores for the 7 student testers were similar for both the interfaces: 72.9 (SD 19.2) for the mobile app and 72.5 (SD 20.7) for the website, equating to *good* usability. The usability issues represented 6 different themes, with the 2 most common being *flexibility and efficiency* and *visibility of system status* (Table 2). Most problems were found in meetings and survey functions. The 2 functions were associated with each other, which meant that if an issue occurred in one, then the other was affected.

**Table 2:** HWPP^a^ Assistant app usability issues reported by student testers (n=7).

App function and usability feedback		Remedied	Usability theme
**Log-in**			
	“Was not automatically logged on website after logging into the app”	Yes	Flexibility and efficiency of use
	“Quicker than expected”	No	Flexibility and efficiency of use
	“Make sure the user is able to retrieve a lost email”	No	Recognition rather than recall
**Meetings**			
	“App does not refresh to meeting tab after I hit ‘create meeting’”	Yes	Visibility of system status
	“Meetings are displayed but I cannot edit them”	No	User control and freedom
	“Change times to a 12-hour [clock]”	Yes	Consistency and standards
**Survey**			
	“Not sure if it posted or not even though it says it was submitted”	Yes	Visibility of system status
	“Surveys I took still say ‘ready’ and do not say ‘taken’.”	Yes	Visibility of system status
	“Slow to load”	Yes	Flexibility and efficiency of use
	“Make sure questions are in order”	Yes	Match between system and the real world
	“Could not go back to the survey and make edits to it”	No	Flexibility and efficiency of use
**Time logs**			
	“Time is not displayed on the app so unclear if it posted”	Yes	Visibility of system status
	“Quicker than expected”	No	Flexibility and efficiency of use
	“Custom time option may be helpful”	No	Flexibility and efficiency of use
	“Confirmation pop up was shown”	No	Visibility of system status
**Chats**			
	“No confirmation...besides my sent message...that [my text] was received, read, or replied to.”	No	Visibility of system status
	“Space to add new contacts...add new chat members”	No	Flexibility and efficiency of use
	“Download new messages faster”	Yes	Flexibility and efficiency of use

^a^HWPP: Healthy Workplace Participatory Program.

#### Download and Log-in

In stage 1, the email addresses of site users were pre-entered, and an initial generic password was set for them. For most tests, the download, log-in, and password change were successful and proceeded more quickly than expected. The functionality issues reported were all fixed by the developer. Testers requested automatic log-in to the accompanying website after logging into the app, but researchers decided that this would compromise confidentiality in a real-world use case where users have to log in using a shared work computer. One tester requested the ability to retrieve a lost email address, but researchers thought it was unlikely that end users would forget their address, and if necessary, they could contact the SHIFT team.

#### Meeting Creation and View

Designing the meetings function for both the computer and the smartphone simultaneously was challenging, as any changes had to be in sync with each other while also ensuring that some features were only on one medium (such as agenda upload on the website). These changes sometimes introduced functionality bugs that testers experienced, which were all fixed by the developer before reaching end users. Several testers requested the ability to edit meetings if they made a mistake, but this change would have required many engineering hours. As there was an existing feature to cancel individual meetings, researchers instead implemented an email feature to inform all study participants (including nonapp users) of changes in meeting date or time.

#### Meeting Survey Submission

Testers’ responses for the survey function centered around data quality concerns such as whether questions were received, in the right order, and were provided for the right meeting type or role. This led to the implementation of a *subject* for meetings and their associated surveys to reduce potential confusion in end users. Other reported issues such as question order, inconsistent push notifications, and slow loading times were also fixed by the developer.

#### Time Log Reporting

All submitted time logs were received in the back end, but sometimes they did not appear under the project time history; this was fixed. Another tester requested more customization of time reported, outside the regular intervals offered by the app, but this was deemed unnecessary because of time and budget constraints.

#### Chat Communications

For the chat function, testers noted that sometimes communication between devices and push notifications were inconsistent and noted that there was no feature to indicate that a text was read or received. One student requested the ability to add new contacts to the chat, outside the project participants, but this was deemed unnecessary as the app is intended only for SHIFT study participants, with user entry by researchers.

### Stage 2: End User Testing

#### Overview

After fixing the issues reported by the students, the app was deployed to end users. The average SUS scores for the 15 end users were similar for the 2 interfaces; scores of 62.33 (SD 20) for the mobile app and 62.5 (SD 17.7) for the website were achieved, equating to *acceptable* usability. The usability issues from end users represented 4 different themes, with the 2 most common being *recognition rather than recall* and *flexibility and efficiency of use* (Table 3)*.* Task scenarios were also revised to target the functions the researchers expected to be most frequently used, as end users mostly had 15-45 minutes to test the app during the deployment meeting.

**Table 3:** HWPP^a^ Assistant app usability issues reported by end users (n=23).

App function and usability feedback			Remedied		Usability theme
**Log-in**					
	“Finger-print option would be helpful”	No		Recognition rather than recall	
	“Due to employer restriction on email access, made it difficult”	Yes		Match between system and the real world	
**Meetings**					
	“Need to be able to edit events”	No		Flexibility and efficiency of use	
	“Email addresses should auto-fill”	No		Recognition rather than recall	
	“Create room location on meeting app”	No		Flexibility and efficiency of use	
**Survey**					
	“Surveys should be associated with meeting”	Yes		Recognition rather than recall	
**Time logs**					
	“Add more minute options”	No		Flexibility and efficiency of use	
	“A bit clunky”	N/A^b^		Aesthetic and minimalist design	
**Other**					
	“People who work with people (clinical, care providers, etc) don’t usually like technical things”	N/A		Match between system and the real world	

^a^HWPP: Healthy Workplace Participatory Program.

^b^N/A: not applicable.

#### Download and Log-in

During testing, the app was still being approved by the university for distribution from the web page, so end users could not download it directly from built-in app stores. This caused some frustration, especially for iOS users, who could not always find the code in their institutional email. This was because of users not having their work email on their phone or the email address being incorrectly entered into the system, so they did not receive the install package. During the password-change task, end users requested the option of entering *the password twice to avoid mistyping it*. To improve efficiency, one user requested a *fingerprint login* function, which has been a rising feature in many apps during this time. However, this was forgone because of the cost and the fact that not all phones would have this feature.

#### Meeting Creation and View

For meeting creation, meetings were not always received by the intended participants. As the users were testing as a group, they were able to look at each other’s phones to see whether anything was submitted. It was at this time that the site research assistants and coaches helped troubleshoot the issue, but there were some issues reported regarding the validation of email addresses. Similar to the students, end users requested the ability to edit a meeting. Room location was requested on the app by one user, but this feature was forgone because of the additional cost of adding the feature and immense restructuring of the back end to accommodate an extra field. In addition, the DTs met consistently, which meant that meeting times and locations did not change frequently.

#### Survey Submission

Some issues reported were similar to those by the student testers, in that end users found that sometimes no survey was offered or received, and there were some screen freezes. Meeting-associated surveys were designed to show up after the meeting end time, but some users found that the survey for the next week showed up early.

#### Time Log Reporting

No functionality issues were reported for the time log function by end users, but there was feedback that it was a bit *clunky*. Similar to the student users, these users requested additional time duration options, but this was forgone because of the cost.

#### Chat Communications

Chats developed an issue that sometimes no text box appeared to type in. With some troubleshooting by the research assistants on site, this issue was resolved but was still reported to the developer.

#### General Feedback and Use

One user provided feedback that people who work for and with people, such as clinical workers or care providers, “don’t usually like technical things.”

For the 4 main functions (meetings, surveys, time logs, and chats), users in both the control and intervention periods spent the most time on meetings, on average, whereas the settings function was used the least. Users were likely to use this function to check if and when there was a meeting occurring. The median time spent on most app functions was generally higher at sites during the control periods than during intervention periods, but there were more visits to each of the pages in the intervention period than during the control periods (Table 4).

Overall, users utilized the app to answer more meeting surveys, set more meetings, and create more time log entries during the intervention period than those at the uncoached sites (Table 5).

**Table 4:** Time spent on HWPP^a^ Assistant app functions by intervention status in the Safety and Health through Integrated, Facilitated Teams study (n=23).

App screen	Control status					Intervention status			
	Visits, n (%)	Minimum screen time, seconds	Maximum screen time, seconds	Median total screen time, seconds	Visits, n (%)		Minimum screen time, seconds	Maximum screen time, seconds	Median total screen time, seconds
Surveys	2 (13)	7	9	8	37 (20)		3.0	349	19
Home	3 (19)	9	98	10	32 (17)		1.0	1561	7.0
Meetings	3 (19)	6	147	130	30 (16)		1.0	899	23.5
Chats	4 (25)	5	10	7	24 (13)		1.0	107	6.5
Profile	1 (6)	29	29	29	18 (10)		2.0	58	5.0
Log-in	1 (6)	43	43	43	14 (7)		18	970	61
Time logs	1 (6)	3	3	3	19 (10)		1.0	299	6.0
Settings	1 (6)	3	3	3	13 (7)		1.0	9.0	2.0

^a^HWPP: Healthy Workplace Participatory Program.

**Table 5:** Number of user entries by HWPP^a^ Assistant app function in the Safety and Health through Integrated, Facilitated Teams study (n=23).

App function	Number of user entries	
	Uncoached period, n (%)	Coached period, n (%)
Surveys	1 (33)	57 (41)
Time logs	0 (0)	42 (30)
Meetings	2 (67)	20 (14)
Chats	0 (0)	11 (8)
Chat threads	0 (0)	9 (6)

^a^HWPP: Healthy Workplace Participatory Program.

## Discussion

### Principal Findings

The primary objective of this study is to describe the logic and sequence of iterative usability testing that informed the development of a mobile app to track a workplace change process evaluation. Testing by students during early iterations of the app was immensely useful for problem discovery and identification of usability problems that would have led to frustration in end users and a potential loss of data in the field. Both student testers and end users mentioned concerns over flexibility and efficiency of use and suggested features related to the app’s ability to recognize information rather than asking users to recall details, but only end users were able to provide important feedback on the match between their real-world occupational context and the app system. App use was aided by the on-site encouragement of the research team, but delays in fixing app issues may have led to initial users normalizing the use of paper data collection alternatives.

Students ranked the usability of the mobile app and website as *good*, whereas the end users ranked the 2 interfaces as *ok*. The most likely reason for this discrepancy was user knowledge of the context and ability to identify problems that students would not have been aware of. However, the difference in SUS scores between students and end users could also be because of student testers being watched during the tests, which may have skewed student responses and scores favorably, otherwise known as the Hawthorne effect. Another possible reason is that students completed these tests on the compensated project work time. Therefore, when problems and errors occurred that affected their ability to perform functions, it may not have caused as much inconvenience as for employees with busy work schedules who were voluntarily taking on extra responsibilities.

Some feature requests by students and end users were based on experiences with other apps or devices’ capabilities. With mobile app development, users’ expectations change over time; some requests may be small, whereas others require large-scale changes. It is difficult to plan for these considerations ahead of time without knowing in advance what software enhancements will become common and will be expected by users [38]. However, using participatory design during the concept stage may be helpful in understanding users’ mental models. Clarifying the vision and needs of the app at an early stage between researchers, software developers, and end users is critical for the success of the app and staying within the budget, which has also been noted by others [38]. Having an additional budget for feature requests may increase users’ satisfaction with the app and potentially increase use.

The higher number of uses of the meetings, surveys, and chat functions by the intervention group may have been because of encouragement by the coach during regularly scheduled meetings, whereas the control groups did not receive the same level of in-person encouragement or support with technical issues. These results are in line with another study’s findings that social influences from colleagues, employers, and health care professionals can exert a strong effect on intention to use a personal health record app in a workplace setting [4].

However, the fit between technical products and the user audience must also be considered, as noted by one end user. All apps must consider the work context, culture, and characteristics of the intended user population [15,39]. When intended for a specific occupational setting, the range of educational levels and experience with new technology may vary greatly among job groups and require strategic choices about whom to design for.

The biggest strength of this study is that our app was uniquely built to document the process outcomes of a workplace change study. The findings and app evaluations from this study provide information on the usefulness of mHealth apps as a data collection method for other researchers conducting workplace interventions.

Testing iteratively was a strength of this study, as it helped the developer pinpoint problem areas, debug across platforms, and inform each stage of development. This resulted in a more refined app for our users during deployment and prevented potential loss of data. The documentation of this iterative process fills a gap noted by others that more usability studies focused on user engagement and product interaction are needed [11].

The use of the SUS is also one of its strengths, as it is a validated instrument for assessing usability, and when combined with the task scenarios, it provided qualitative feedback from users as well. This mixed methods approach provided multidimensional information to customize the app for both the researchers’ and target users’ needs. Future studies looking to develop an app with a similar purpose will be able to build upon what we have done and avoid potential pitfalls that may result in substantial project delays.

### Limitations

One weakness of our study is that the small end user sample limited the ability to stratify by demographics, site, or other variables of interest.

The use of Google Analytics, although useful as another measure of app use, did not capture some user visits. We also did not ask specific questions on reasons for adoption and reasons for attrition, which might have provided additional information on why some users dropped out early and some dropped out later. However, there seemed to be a substantial shift in app users opting for paper surveys after some fixes took longer than expected. This delay was because of a change in the developers hired for this research project to maintain the app, which required onboarding time. Although not covered in this paper, this study depended heavily on the paper duplications of the app functions not only for nonapp users but also for when the app encountered issues, and this should be expected for the development of apps for assisting with data collection in a workplace intervention study. Future work will involve the analysis of the process data that were collected through the app for the SHIFT study.

### Conclusions

End users deemed our process evaluation mobile app to be of acceptable usability, thanks to the student testers identifying a number of bugs and errors that could be fixed before deployment to our study population. Researchers looking to develop an app for a similar purpose would benefit from early and iterative user testing. Understanding user standards for a usable app and budgeting to keep up with the pace of other apps’ features could improve overall satisfaction and acceptability.

## Abbreviations

DT: Design Team
HWPP: Healthy Workplace Participatory Program
mHealth: mobile health
SC: Steering Committee
SHIFT: Safety and Health through Integrated, Facilitated Teams
SUS: System Usability Scale

## References

[1] Butterfoss FD (2006). Process evaluation for community participation. Annu Rev Public Health.

[2] Steckler A, Linnan L (2002). Process Evaluation for Public Health Interventions and Research.

[3] Department of Health and Human Services (2013). Mobile health: technology and outcomes in low and middle income countries (R21). National Institutes of Health.

[4] Park HS, Kim KI, Soh JY, Hyun YH, Jang SK, Lee S, Hwang GY, Kim HS (2020). Factors influencing acceptance of personal health record apps for workplace health promotion: cross-sectional questionnaire study. JMIR Mhealth Uhealth.

[5] Jimenez P, Bregenzer A (2018). Integration of eHealth tools in the process of workplace health promotion: proposal for design and implementation. J Med Internet Res.

[6] Mattila E, Orsama A, Ahtinen A, Hopsu L, Leino T, Korhonen I (2013). Personal health technologies in employee health promotion: usage activity, usefulness, and health-related outcomes in a 1-year randomized controlled trial. JMIR Mhealth Uhealth.

[7] Jaworski B, Kohli AK, Sahay A (2000). Market-driven versus driving markets. J Acad Mark Sci.

[8] Ericsson K, Hoffman R, Kozbelt A, Williams A (2018). The Cambridge Handbook of Expertise and Expert Performance.

[9] User-centered design basics. U.S. Department of Health & Human Services.

[10] Usability testing. U.S. Department of Health & Human Services.

[11] Georgsson M, Staggers N (2016). Quantifying usability: an evaluation of a diabetes mHealth system on effectiveness, efficiency, and satisfaction metrics with associated user characteristics. J Am Med Inform Assoc.

[12] Hilliard ME, Hahn A, Ridge AK, Eakin MN, Riekert KA (2014). User preferences and design recommendations for an mhealth app to promote cystic fibrosis self-management. JMIR Mhealth Uhealth.

[13] Hudson W (2009). Reduced empathizing skills increase challenges for user-centered design. Proceedings of the SIGCHI Conference on Human Factors in Computing Systems.

[14] Card sorting. U.S. Department of Health & Human Services.

[15] Brown M, Hooper N, Eslambolchilar P, John A (2020). Development of a web-based acceptance and commitment therapy intervention to support lifestyle behavior change and well-being in health care staff: participatory design study. JMIR Form Res.

[16] Zhang D, Adipat B (2005). Challenges, methodologies, and issues in the usability testing of mobile applications. Int J Hum-Comput Int.

[17] Moran K (2019). Usability testing 101. Nielsen Norman Group.

[18] Hirschheim R (1983). Assessing participative systems design: some conclusions from an exploratory study. Inf Manag.

[19] Pilemalm S, Timpka T (2008). Third generation participatory design in health informatics - making user participation applicable to large-scale information system projects. J Biomed Inform.

[20] Reporting usability test results. U.S. Department of Health & Human Services.

[21] Zapata BC, Fernández-Alemán JL, Idri A, Toval A (2015). Empirical studies on usability of mHealth apps: a systematic literature review. J Med Syst.

[22] Ma X, Yan B, Chen G, Zhang C, Huang K, Drury J (2012). A toolkit for usability testing of mobile applications. Mobile Computing, Applications, and Services.

[23] Nielsen J, Landauer T (1993). A mathematical model of the finding of usability problems. Proceedings of the INTERACT'93 and CHI'93 Conference on Human Factors in Computing Systems.

[24] Eysenbach G, CONSORT-EHEALTH Group (2011). CONSORT-EHEALTH: improving and standardizing evaluation reports of web-based and mobile health interventions. J Med Internet Res.

[25] Goyal S, Morita PP, Picton P, Seto E, Zbib A, Cafazzo JA (2016). JMIR Mhealth Uhealth.

[26] De Vito Dabbs A, Myers BA, Mc Curry KR, Dunbar-Jacob J, Hawkins RP, Begey A, Dew MA (2009). User-centered design and interactive health technologies for patients. Comput Inform Nurs.

[27] Boudreaux ED, Fischer AC, Haskins BL, Zafar ZS, Chen G, Chinai SA (2016). Implementation of a computerized screening inventory: improved usability through iterative testing and modification. JMIR Hum Factors.

[28] Krebs P, Duncan DT (2015). Health app use among US mobile phone owners: a national survey. JMIR Mhealth Uhealth.

[29] Bender MS, Choi J, Arai S, Paul SM, Gonzalez P, Fukuoka Y (2014). Digital technology ownership, usage, and factors predicting downloading health apps among Caucasian, Filipino, Korean, and Latino Americans: The digital link to health survey. JMIR Mhealth Uhealth.

[30] Punnett L, Nobrega S, Zhang Y, Rice S, Gore R, Kurowski A, SHIFT Project Research Team (2020). Safety and Health through Integrated, Facilitated Teams (SHIFT): stepped-wedge protocol for prospective, mixed-methods evaluation of the Healthy Workplace Participatory Program. BMC Public Health.

[31] Robertson M, Henning R, Warren N, Nobrega S, Dove-Steinkamp M, Tibirica L, Bizarro A, CPH-NEW Research Team (2013). The Intervention Design and Analysis Scorecard: a planning tool for participatory design of integrated health and safety interventions in the workplace. J Occup Environ Med.

[32] Chin W, Kurowski A, Chen G, Gore R, Punnett L (2019). Enhancing the usability of a mobile app for process evaluation in a participatory ergonomics healthcare intervention. Proceedings of the 20th Congress of the International Ergonomics Association (IEA 2018).

[33] Dumas J, Redish J (1999). Practical Guide to Usability Testing.

[34] Brooke J (1996). SUS: a 'Quick and Dirty' usability scale. Usability Evaluation In Industry.

[35] Bangor A, Kortum P, Miller J (2009). Determining what individual SUS scores mean: adding an adjective rating scale. J Usability Stud.

[36] Sauro J (2011). A Practical Guide to the System Usability Scale: Background, Benchmarks & Best Practices.

[37] Nielsen J (1994). Heuristic Evaluation.

[38] Roth WR, Vilardaga R, Wolfe N, Bricker JB, McDonell MG (2014). Practical considerations in the design and development of smartphone apps for behavior change. J Contextual Behav Sci.

[39] Muuraiskangas S, Harjumaa M, Kaipainen K, Ermes M (2016). Process and effects evaluation of a digital mental health intervention targeted at improving occupational well-being: lessons from an intervention study with failed adoption. JMIR Mental Health.

